# Pancreatic neuroendocrine tumor with complete replacement of the pancreas by serous cystic neoplasms in a patient with von Hippel-Lindau disease: a case report

**DOI:** 10.1186/s40792-017-0381-4

**Published:** 2017-09-25

**Authors:** Shimpei Maeda, Fuyuhiko Motoi, Shuhei Oana, Kyohei Ariake, Masamichi Mizuma, Takanori Morikawa, Hiroki Hayashi, Kei Nakagawa, Takashi Kamei, Takeshi Naitoh, Michiaki Unno

**Affiliations:** 1 Department of Surgery, Tohoku University Graduate School of Medicine, 1-1 Seiryo-machi, Aoba-ku, Sendai, Miyagi 980-8574 Japan; 2 Division of Gastroenterology, Department of Internal Medicine, Iwate Medical University, 19-1 Uchimaru, Morioka, Iwate, 020-8505 Japan

**Keywords:** Pancreatic neuroendocrine tumor, Serous cystic neoplasm, von Hippel-Lindau disease, Total pancreatectomy, Pancreatic insufficiency

## Abstract

**Background:**

von Hippel-Lindau disease is a dominantly inherited multi-system syndrome with neoplastic hallmarks. Pancreatic lesions associated with von Hippel-Lindau include serous cystic neoplasms, simple cysts, and neuroendocrine tumors. The combination of pancreatic neuroendocrine tumors and serous cystic neoplasms is relatively rare, and the surgical treatment of these lesions must consider both preservation of pancreatic function and oncological clearance. We report a patient with von Hippel-Lindau disease successfully treated with pancreas-sparing resection of a pancreatic neuroendocrine tumor where the pancreas had been completely replaced by serous cystic neoplasms, in which pancreatic function was preserved.

**Case presentation:**

A 39-year-old female with von Hippel-Lindau disease was referred to our institution for treatment of a pancreatic neuroendocrine tumor. Abdominal computed tomography demonstrated a well-enhanced mass, 4 cm in diameter in the tail of the pancreas, and two multilocular tumors with several calcifications, 5 cm in diameter, in the head of the pancreas. There was complete replacement of the pancreas by multiple cystic lesions with diameters ranging from 1 to 3 cm. Magnetic resonance cholangiopancreatography showed innumerable cystic lesions on the whole pancreas and no detectable main pancreatic duct. Endoscopic ultrasound-guided fine-needle aspiration of the mass in the pancreatic tail showed characteristic features of a neuroendocrine tumor. A diagnosis of pancreatic neuroendocrine tumor in the tail of the pancreas and mixed-type serous cystic neoplasms replacing the whole pancreas was made and she underwent distal pancreatectomy while avoiding total pancreatectomy. The stump of the pancreas was sutured as firm as possible using a fish-mouth closure. The patient made a good recovery and was discharged on postoperative day 9. She is currently alive and well with no symptoms of endocrine or exocrine pancreatic insufficiency 8 months after surgery.

**Conclusions:**

A pancreas-sparing resection should be considered for patients with pancreatic neuroendocrine tumors and complete cystic replacement of the pancreas to preserve quality of life after surgery.

## Background

von Hippel-Lindau (VHL) disease is a dominantly inherited multi-system syndrome with hallmark neoplastic features [1]. Because of the diverse multi-system effects of this disease, careful and selective treatment should be provided to individuals. Pancreatic lesions associated with VHL include serous cystic neoplasms (SCNs), simple cysts, and pancreatic neuroendocrine tumors (PNETs). VHL-associated SCNs usually manifest as multifocal discrete lesions and sometimes with diffuse pancreatic involvement. Further, they may also present with a concomitant PNET. The choice of surgical treatment of these lesions must consider both preservation of pancreatic function and oncological clearance. Herein, we report a case of a patient with VHL, successfully treated by curative resection and distal pancreatectomy for PNET with completely replaced pancreas by SCNs, in which pancreatic function could be preserved.

## Case presentation

**Fig. 1:**
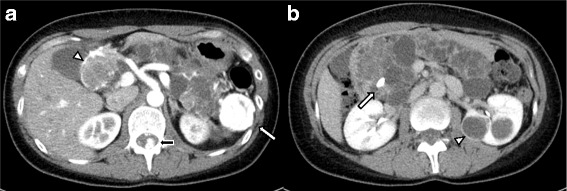
**a** Abdominal computed tomography showed a well-enhanced mass, 4 cm in diameter, in the tail of the pancreas (*white arrow*) and multilocular tumors in the head of the pancreas (*arrowhead*). One small, enhancing focus was seen in the spinal canal at the L1 level (*black arrow*). **b** The pancreas was completely replaced with mixed microcystic and macrocystic serous cystic neoplasms. Several calcifications of the pancreas (*arrow*) and renal cell carcinoma (*arrowhead*) were observed

**Fig. 2:**
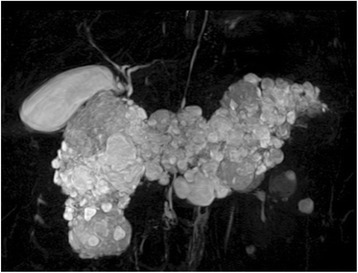
Magnetic resonance cholangiopancreatography showed innumerable cystic lesions of the whole pancreas and no detectable main pancreatic duct

**Fig. 3:**
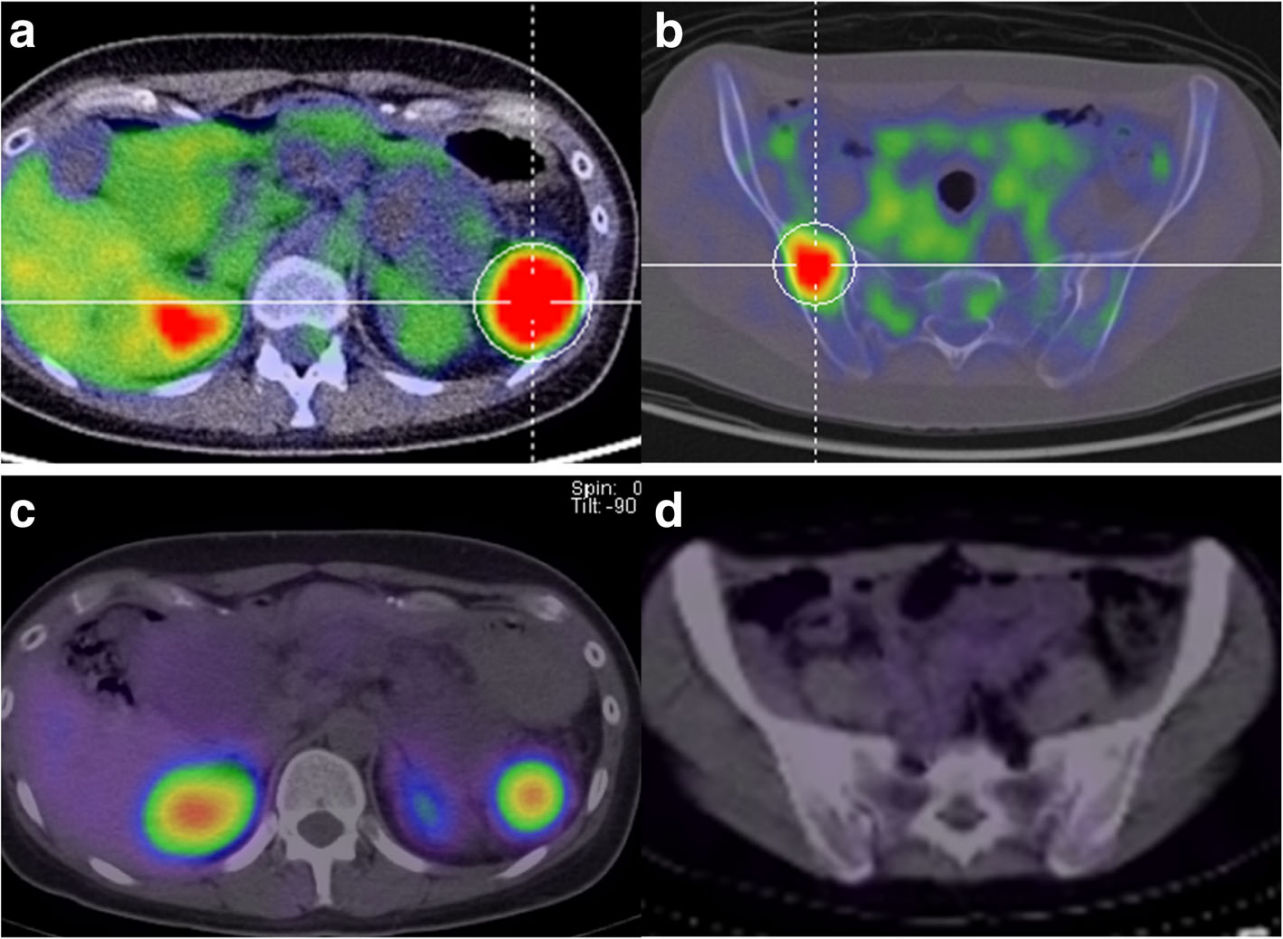
Fluorodeoxyglucose-positron emission tomography (FDG-PET) demonstrated the accumulation of FDG in a solid tumor in the tail of the pancreas (**a**) and the right ilium (**b**) with a maximum standardized uptake value of 9.5 and 9.4, respectively. Somatostatin receptor scintigraphy showed accumulation of radioactivity in the tumor in the pancreatic tail (**c**) but not in the right ilium (**d**)

**Fig. 4:**
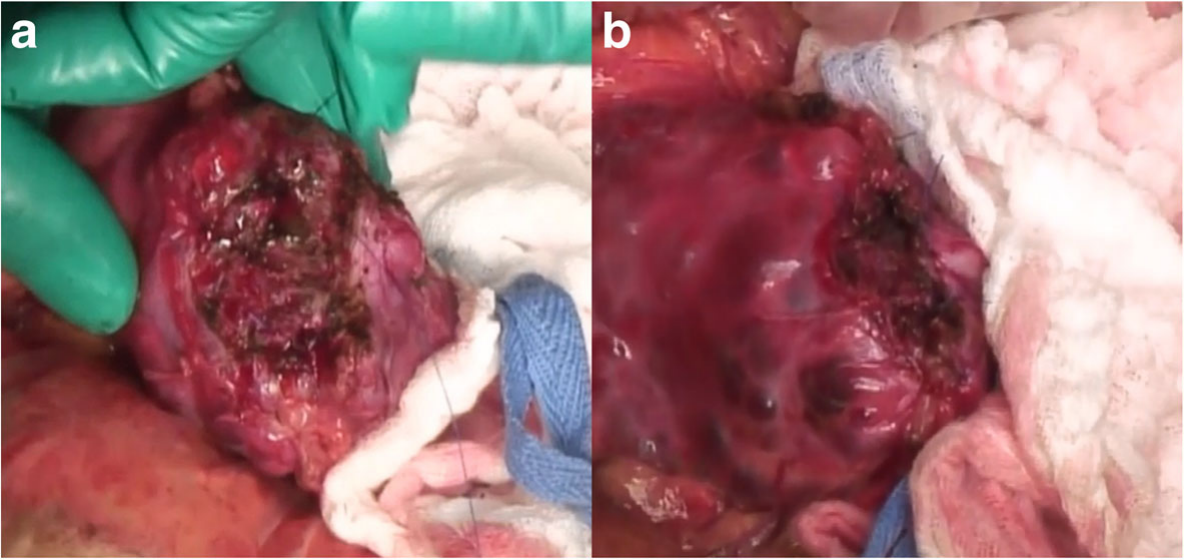
Intraoperative photographs demonstrating the stump of the pancreas before (**a**) and after (**b**) suturing

A 39-year-old female with VHL disease was referred to our institution for treatment of PNET associated with multiple cystic lesions of the whole pancreas detected by follow-up imaging. She had a history of hemangioblastomas of the cerebellum and retina treated surgically 9 and 16 years before, respectively, and renal cell carcinomas followed with careful surveillance. She had no relevant family history. There were no complaints involving abdominal discomfort, pain, jaundice, or steatorrhea. Laboratory tests showed no abnormal data. Blood glucose, hemoglobin A1c, and the level of amylase were normal, and tumor markers, including cancer antigen-19-9 (22.4 U/ml) and carcinoembryonic antigen (3.5 ng/ml), were within normal limits. Abdominal computed tomography (CT) demonstrated a well-enhanced mass, 4 cm in diameter, in the tail of the pancreas; two multilocular tumors with several calcifications, 5 cm in diameter, in the head of the pancreas; and the replacement of whole pancreas by multiple cystic lesions with diameters of 1−3 cm (Fig. 1). One small, round, enhancing focus was seen in the spinal canal at the L1 level which was thought to be a hemangioblastoma. Endoscopic ultrasound-guided fine-needle aspiration of the mass in the pancreatic tail showed features of PNET. Magnetic resonance cholangiopancreatography showed innumerable cystic lesions of the whole pancreas and no detectable main pancreatic duct (Fig. 2). Upon 18F-fluorodeoxyglucose-positron emission tomography (FDG-PET) analysis, intense FDG uptake was seen in the tumor in the pancreatic tail and the right ilium (Fig. 3a, b). Somatostatin receptor scintigraphy showed accumulation of radioactivity in the tumor in the pancreatic tail but not in the right ilium (Fig. 3c, d). CT-guided needle biopsy revealed the iliac lesion to be a marginal zone lymphoma, which did not require immediate treatment. A diagnosis of PNET in the tail of the pancreas and mixed microcystic and macrocystic SCNs replacing the whole pancreas was made and she was offered surgical treatment. Written informed consent for a total pancreatectomy was obtained because it was unclear whether it was safe to transect the pancreas completely replaced with cystic lesions. While transecting the pancreas between the cysts using THUNDERBEAT™ (Olympus, Japan), an integrated energy device, a distal pancreatectomy was performed successfully. The main pancreatic duct was invisible, and the cut edge of the pancreas was sutured by 4-0 absorbable monofilament using a fish-mouth closure as firm as possible (Fig. 4). The total operation time was 269 min, and the estimated blood loss was 442 ml. The patient made a good recovery and was discharged on postoperative day 9. She is currently alive and well with no symptoms of endocrine or exocrine pancreatic insufficiency 8 months after surgery.

**Fig. 5:**
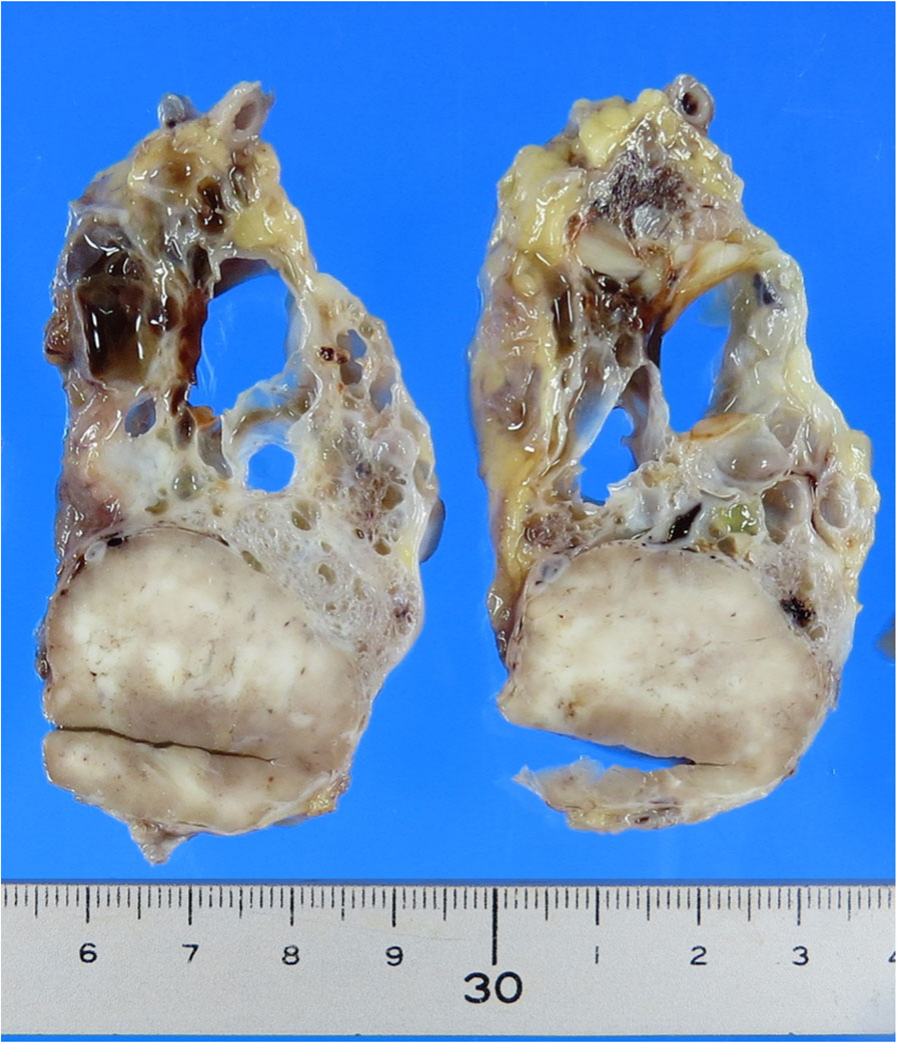
Gross photograph of a pancreatic neuroendocrine tumor in the tail and the surrounding serous cyst neoplasms

**Fig. 6:**
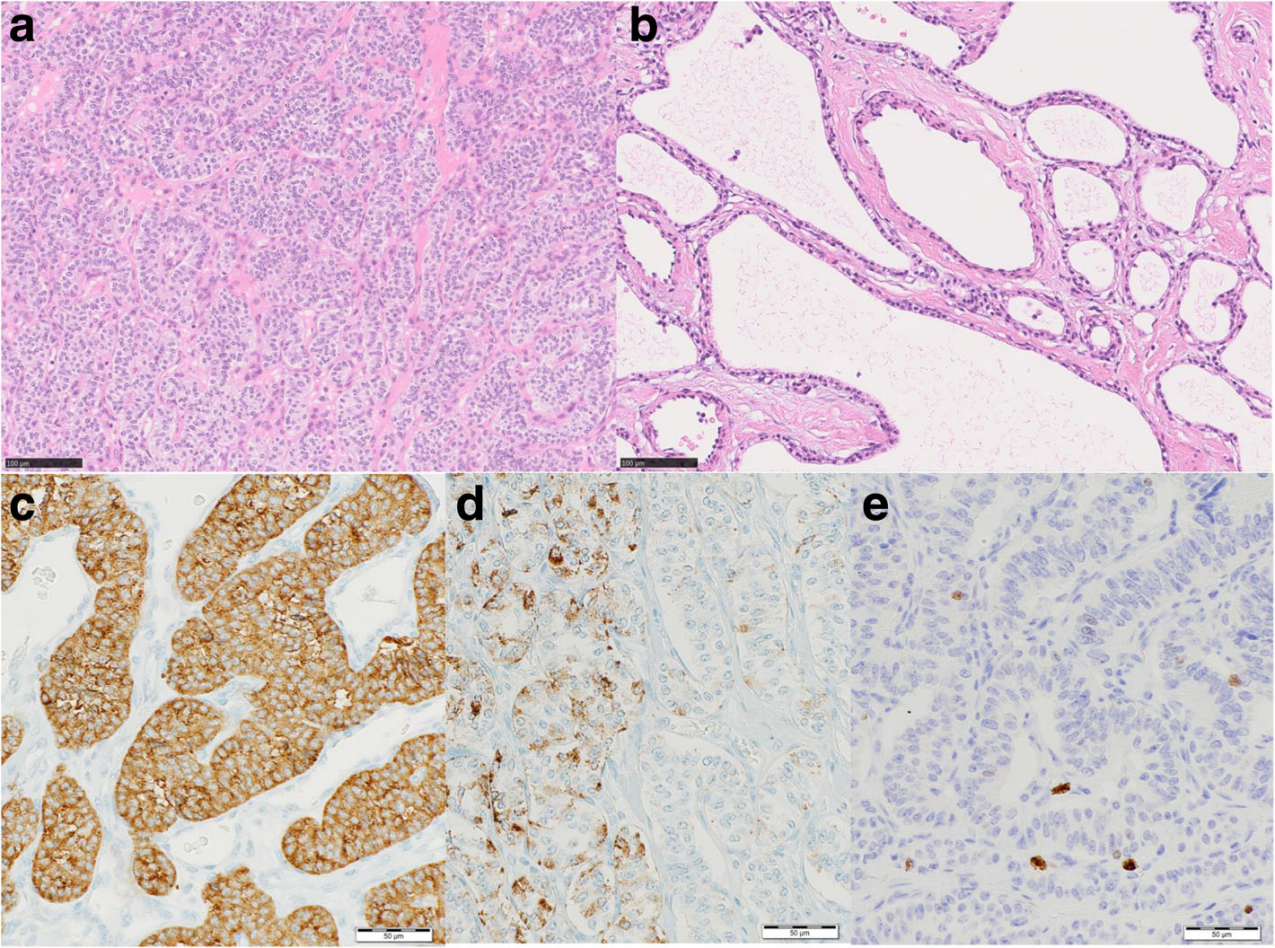
Microscopically, the tumor in the tail of the pancreas showed a trabecular architecture with salt-and-pepper chromatin, eosinophilic cytoplasm, and abundant microvasculature (**a**). The cysts were serous cystadenomas lined with glycogen-rich cuboidal to flattened epithelial cells with clear cytoplasm and uniform round nuclei (**b**). Immunohistochemically, the tumor cells stained positive for synaptophysin (**c**) and focally positive for chromogranin A (**d**). The Ki-67 labeling index was 1% (**e**)

Macroscopically, a grayish-white solid mass of 3 cm in diameter was noted in the tail of the pancreas. The surrounding pancreas was replaced by numerous cysts of varying sizes (Fig. 5). Histologic examination of the solid lesion demonstrated a well-differentiated grade 1 PNET, showing a trabecular architecture with salt-and-pepper chromatin, eosinophilic cytoplasm, and abundant microvasculature (Fig. 6). Immunohistochemically, the tumor cells stained positive for synaptophysin and focally positive for chromogranin A. The Ki-67 labeling index was 1%, and the mitotic count was 0/10 high power fields. No vascular invasion was present, and the lymph nodes examined were negative for metastasis. The cysts were serous cystadenomas lined with glycogen-rich cuboidal to flattened epithelial cells with clear cytoplasm and uniform round nuclei.

## Discussion

To the best of our knowledge, no reports of a patient undergoing a pancreas-sparing resection for PNET with complete cystic replacement of the pancreas have been described in the literature. The pancreas has endocrine and exocrine functions that are essential for life. Total pancreatectomy leads to pancreatic insufficiency, resulting in brittle diabetes, malabsorption, hepatic steatosis, and decreased quality of life [2]. In our case, the postoperative course was uneventful with no symptoms of pancreatic insufficiency. Surgeons should try to avoid a total pancreatectomy if oncologically appropriate and technically possible.

With an incidence of approximately one in 36,000 live births, VHL disease is an autosomal dominant multi-system neoplastic syndrome resulting from a germline mutation of the VHL tumor suppressor gene on the short arm of chromosome 3 (3p25-26) [1, 3]. Affected individuals are at risk of developing various benign and malignant tumors of the central nervous system and retina, kidney, adrenal glands, reproductive adnexal organs, and pancreas. In general, these patients should be managed using an organ-sparing strategy whenever possible [4]. Pancreatic lesions associated with VHL can be found in 49−77% of patients, including cysts (47−70%), SCNs (9−11%), and PNETs (9−17%). A combination of PNET and SCN is relatively rare, with incidence ranging from 1−2% in patients with VHL disease [5–9].

SCN is a rare tumor, accounting for 1−2% of all exocrine pancreatic tumors, and is characterized by glycogen-rich cuboidal epithelial cells with uniform round nuclei and dense, homogenous chromatin [10–12]. SCN is benign in nearly all cases. Recently, Jais and coauthors reported that in a multinational study, the proportion of serous cystadenocarcinoma was less than 0.1% and lower than commonly described in the literature (1-3%) [13]. Surgical treatment for SCN should be carried out only in a minority of patients, i.e., those with uncertain diagnoses, cases with significant related symptoms, or exceptionally when there is concern regarding malignancy [13, 14].

On the other hand, PNETs can metastasize. Metastasis is reported to be found in 8−13% of PNETs that develop in patients with VHL [5–9]. Therefore, PNETs should be managed with surgical intervention. Libutti and colleagues recommended resecting VHL-associated PNETs if the tumor is greater than 3 cm and if the tumor is greater than 2 cm in the head of the pancreas to allow the chance of enucleation rather than resection of the head of the pancreas [4]. Other factors associated with an increased risk of malignancy include rapid tumor doubling time of less than 500 days [7]. In addition, de Mestier et al. reported that VHL-associated PNETs which were less than 1.5 cm in diameter and deliberately left in place did not tend to progress after 10 years of median follow-up. This suggests pancreas-sparing surgical management may be considered in this patient population [15]. In our case, the size of the PNET was 4 cm at diagnosis, which met the criteria for resection.

Only three cases of PNET with complete cystic replacement of the pancreas have been described in the literature (Table 1) [16–19]. Baek et al. and Jung et al. reported on the same patient. Cases in which the lesions showed multifocal but not complete replacement and in which detailed data were not available were excluded. Of four cases including our patient, patients ranged in age from 29 to 67 years, and all cases were female. Two patients had VHL disease. Our case was the only one where the PNET was located in the tail of the pancreas. As anticipated, the main pancreatic duct was invisible, and it may have been difficult to close or anastomose the main pancreatic duct by an ordinary procedure. In this case, the cut edge of the pancreas, mainly the pancreatic capsule, was sutured to close like a fish mouth paying attention to not rupture the cystic lesions [20], resulting in a successful postoperative course. Development of the integrated energy device may also contribute to the success of this procedure including, but not limited to, sealing the potentially invisible main pancreatic duct. However, as these cases are extremely rare, there remains debate regarding safety and outcomes. Further information will be required to clarify this issue.

**Table 1:** Patients with pancreatic neuroendocrine tumor and complete replacement of the pancreas by serous cystic neoplasms

Authors (year)	Patient’s sex/age	Location of PNET	VHL association	Operation
Kim et al. (1997)	F/67	Head	Absent	Total pancreatectomy
Baek et al. (2000) Jung et al. (2001)	F/29	Head	Present	Total pancreatectomy
Agarwal et al. (2009)	F/35	Head	Absent	Total pancreatectomy
Our case	F/39	Tail	Present	Distal pancreatectomy

*PNET* pancreatic neuroendocrine tumor, *VHL* von Hippel-Lindau

## Conclusions

A pancreas-sparing resection should be carried out in patients with PNET and complete cystic replacement of the pancreas to maintain quality of life after surgery.

## Abbreviations

CT: Computed tomography
FDG-PET: 18F-fluorodeoxyglucose-positron emission tomography
PNET: Pancreatic neuroendocrine tumor
SCN: Serous cystic neoplasm
VHL: von Hippel-Lindau
